# Germline multigene panel testing in acute and chronic pancreatitis

**DOI:** 10.1371/journal.pone.0307076

**Published:** 2024-08-22

**Authors:** Mitchell L. Ramsey, Brandie Heald, Yevgeniya Gokun, Josie Baker, J. Royce Groce, Samuel Han, Phil A. Hart, Somashekar G. Krishna, Luis F. Lara, Peter J. Lee, Georgios I. Papachristou, Rachel Pearlman, Sarah Poll, Maegan E. Roberts, Peter P. Stanich

**Affiliations:** 1 Division of Gastroenterology, Hepatology, and Nutrition, The Ohio State University Wexner Medical Center, Columbus, Ohio, United States of America; 2 Medical Affairs, Invitae Corporation, San Francisco, California, United States of America; 3 Department of Biomedical Informatics, Center for Biostatistics, The Ohio State University Wexner Medical Center, Columbus, Ohio, United States of America; 4 Division of Human Genetics, The Ohio State University Wexner Medical Center, Columbus, Ohio, United States of America; Universita degli Studi di Roma Tor Vergata, ITALY

## Abstract

**Background/Objectives:**

Germline genetic testing is recommended for younger patients with idiopathic pancreatitis but there has been a lack of consensus in recommendations for those over age 35. We aimed to analyze the results of genetic testing among subjects of varying ages.

**Methods:**

Individuals who underwent germline multigene testing for pancreatitis susceptibility genes (*CASR*, *CFTR*, *CPA1*, *CTRC*, *PRSS1*, *SPINK1*) through a large commercial laboratory between 2017 and 2022 were included. Test results and information collected from test requisition forms were evaluated. Multivariable logistic regression models were performed to identify factors associated with a positive pancreatitis panel (pathogenic, likely pathogenic, and/or increased risk variants) in pancreatitis-related genes.

**Results:**

Overall, 2,468 subjects with primary indication of acute pancreatitis (AP) (n = 401), chronic pancreatitis (CP) (n = 631), pancreatic cancer (n = 128), or other indications (n = 1,308) completed germline testing. Among patients with AP or CP, the prevalence of any positive result for those <35 versus ≥35 years of age was 32.1% and 24.5% (p = 0.007), and the prevalence of a clinically meaningful result was 10.8% and 5.4%, respectively (p = 0.001). Positive family history of pancreatitis was associated with increased odds ratio (OR) of 8.59 (95% confidence interval (CI) 2.92–25.25) for a clinically significant panel result while each 5-year increase in age at test completion had lower odds (OR 0.89, 95% CI 0.83–0.95).

**Conclusions:**

The highest prevalence of pathogenic variants is seen in younger individuals with a positive family history of pancreatitis. However, clinically meaningful results are identified in older subjects, suggesting that genetic counseling and testing should be considered for all age groups.

## Introduction

The risk of developing acute pancreatitis (AP) and chronic pancreatitis (CP) involves an interaction between environmental and genetic factors [1, 2]. The roles that pancreatitis-related genes play in the development of CP have informed our understanding of the pathophysiology of CP [3, 4]. Pathogenic and likely pathogenic variants (PVs) in some genes (i.e., *PRSS1*) may lead to CP in the absence of environmental risk factors, while PVs in other genes (i.e., *CLDN*) may lower the threshold for developing CP among patients with environmental exposures [5, 6]. Furthermore, some pancreatitis-related genes (i.e., *CFTR*) display complex inheritance patterns and low penetrance, such that identifying high risk patients for selective referrals for genetic testing is challenging [7].

Among patients with CP, the prevalence of PVs in pancreatitis-related genes has been reported from 15% to 80% [8–12]. This wide variation is attributed to differences in subject demographics, referral practices, the number of genes tested, genetics laboratory techniques, and, in the case of *CFTR* and *SPINK1*, the carrier frequency in the population [8, 13]. Furthermore, the majority of these studies were small, single-center experiences, and multicenter series are rarely encountered [14]. This wide variation in reported prevalence presents a challenge for providing a risk assessment during pre-test genetic counseling [15, 16]. Clinical guidelines recommend germline multigene panel testing for patients younger than 35 years of age with unexplained CP, but recommendations for patients older than 35, patients with some environmental exposures (i.e., alcohol use), and patients with recurrent AP lack consensus, possibly related to the perceived lower prevalence of PVs [17–20].

We sought to describe the results of a germline multigene pancreatitis panel in a population of patients referred for testing through a commercial laboratory. We hypothesized that the distribution of positive results would be different among disease groups and according to age at the time of testing.

## Methods

The Institutional Review Board (IRB) at The Ohio State University and at Invitae^®^ approved this study prior to initiation. Both IRBs waived the requirement for written informed consent due to the minimal risk of using de-identified data. Individuals of all ages who completed the Chronic Pancreatitis Panel for any indication at a large commercial laboratory (Invitae, San Francisco, California, USA) between January 1, 2017 and March 20, 2022 were eligible for inclusion. The de-identified dataset was created on March 21, 2022. Subject demographics, indication for testing, and family and medical history were obtained from test requisition forms submitted at the time of test ordering. Additional clinical information (i.e., alcohol use, imaging or lab characteristics) is not provided on test requisition forms. A positive family history of pancreatitis includes either AP or CP in any relative. Similarly, a positive family of pancreatic cancer includes pancreatic cancer in any relative. Self-reported race/ethnicity was recorded as ‘White’, ‘Non-White’ (included ‘Black/African American’, ‘Asian’, ‘More than One Race,’ and ‘Other’) and ‘Unknown/not provided’ categories.

When multiple indications for testing were recorded, then the group assignments were attributed according to the following hierarchy: CP, AP, pancreatic cancer, or other when none of the diagnostic codes of interest were present. Accordingly, if a subject carried a diagnosis of both AP and CP, then the subject was included in the CP group. Subjects were included in the “other” group if no diagnostic codes for AP, CP, or pancreatic cancer were listed on the test requisition. Thus, this “other” group is heterogeneous, and could include individuals with pancreatitis but incomplete test requisition forms or individuals who underwent testing for another indication. Using this classification algorithm, subjects were sorted into the following groups: pancreatic cancer (n = 128), AP (n = 401), CP (n = 631), or other (n = 1308).

### Multigene panel composition

All individuals underwent testing with the Invitae Chronic Pancreatitis Panel, which is a germline multigene panel including *CASR* (NM_000388.3), *CFTR* (NM_000492.3), *CTRC* (NM_001868.3), *PRSS1* (NM_002769.4), and *SPINK1* (NM_003122.4) [21]. In July 2019, *CPA1* (NM_001868.4) was added to the multigene pancreatitis panel such that individuals tested after this date were assessed for a minimum of these 6 genes. The Invitae laboratory is certified for clinical use and patient reporting under the Centers for Medicare and Medicaid Services’ Clinical Laboratory Improvement Amendments (CLIA). Genomic DNA was extracted from whole blood, buccal swabs, or saliva, and next-generation sequencing was performed to analyze deletions, duplications, and single nucleotide substitutions through full gene sequencing, as previously described [22, 23].

### Sequencing and variant interpretation

All variants, whether single nucleotide variants or structural variations, were interpreted according to a refinement of the American College of Medical Genetics and Genomics criteria [24]. Subjects with at least one PV and/or increased risk allele were considered to have a positive test result. Subjects with a single PV in a recessive gene (*CFTR*, *SPINK1*) were considered positive, as has been done previously [25–28]. Variants of uncertain significance (VUS) were classified as negative test results. Positive panel results with implications for clinical management include heterozygous *PRSS1*, biallelic *CFTR*, biallelic *SPINK1*, or a combination of two or more positive results (i.e., *CFTR* and *SPINK1*) [7].

### Statistical analysis

Descriptive statistics were displayed with frequencies and percentages for categorical variables with means along with standard deviations (or medians along with interquartile ranges) for continuous variables. Chi-square, Fisher’s exact, and Kruskal Wallis Tests were used to assess differences based on the primary indication for testing. Univariate logistic regressions predicting the primary outcome (positive pancreatitis-related gene) were completed for each independent variable and p-values from Wald-Chi-Square Tests were reported.

Multivariable binary logistic regression for the outcome of a positive pancreatitis panel was completed, including AP and CP subjects with adjustment for age, sex, race, year of testing, primary indication for testing, and family history of pancreatitis and pancreatic cancer. Year of testing was included in order to determine whether changes in referral patterns for genetic testing throughout the study period was associated with the frequency of identifying a positive result. A second multivariable binary logistic regression for the outcome of positive pancreatitis panel with implications for clinical management was completed, including AP and CP subjects with adjustment for age, sex, race/ethnicity, primary indication for testing, and family history of pancreatitis. Year of testing and family history of pancreatic cancer were not included in the second multivariable logistic regression due to a smaller number of subjects in this model. These two models were performed first with age as a continuous variable and then treating age as binary variable comparing those who are 35 or older versus those who were younger than 35 years of age. All analyses were performed using SAS v9.4 (SAS Institute; Cary, NC). Statistical significance was defined as two-sided alpha < 0.05.

## Results

### Study population

A total of 2,468 subjects met study criteria and completed germline genetic testing with the multigene pancreatitis panel. The sex, age at time of testing, family histories, and results of germline testing were different between pancreatitis phenotypes, notably including younger age in the AP group, higher prevalence of positive pancreatitis family history in the AP and CP groups, and higher prevalence of PVs in pancreatitis-associated genes in the AP and CP groups (Table 1).

**Table 1:** Demographics and characteristics of subjects who completed germline testing with a multigene pancreatitis panel from 2017 to 2022.

	Acute Pancreatitis (n = 401)	Chronic Pancreatitis (n = 631)	Pancreatic Cancer (n = 128)	Other (n = 1308)	P-Value
**Sex**					<0.0001
Female	223 (55.6%)	374 (59.3%)	70 (54.7%)	903 (69.0%)	
Male	178 (44.4%)	257 (40.7%)	58 (45.3%)	405 (31.0%)	
**Age at Testing**					
Mean (SD)	33.0 (17.7)	38.3 (19.2)	62.9 (11.8)	47.8 (19.7)	<0.0001
Median (IQR)	33 (18–45)	39 (21–52)	63.5 (56.5–71)	50 (34–64)	
**Year of Testing**					<0.0001
2017	39 (9.7%)	55 (8.7%)	5 (3.9%)	53 (4.1%)	
2018	50 (12.5%)	105 (16.6%)	13 (10.2%)	135 (10.3%)	
2019	73 (18.2%)	148 (23.5%)	27 (21.1%)	256 (19.6%)	
2020	99 (24.7%)	159 (25.2%)	34 (26.6%)	353 (27.0%)	
2021	117 (29.2%)	137 (21.7%)	41 (32.0%)	444 (33.9%)	
2022	23 (5.7%)	27 (4.3%)	8 (6.3%)	67 (5.1%)	
**Self-Reported Race/Ethnicity**					0.12
White	302 (75.3%)	436 (69.1%)	109 (85.2%)	1046 (80.0%)	
Black/African American	22 (5.5%)	35 (5.6%)	6 (4.7%)	59 (4.5%)	
Asian	17 (4.2%)	19 (3.0%)	5 (3.9%)	47 (3.6%)	
More than one race	14 (3.5%)	26 (4.1%)	5 (3.9%)	69 (5.3%)	
Other	17 (4.2%)	8 (1.3%)	0 (0%)	30 (2.3%)	
Unknown/not provided	29 (7.2%)	107 (17.0%)	3 (2.3%)	57 (4.4%)	
**Family History of Pancreatitis**					<0.0001
Positive	54 (13.5%)	80 (12.7%)	6 (4.7%)	122 (9.3%)	
Negative	53 (13.2%)	78 (12.4%)	42 (32.8%)	362 (27.7%)	
Not provided	294 (73.3%)	473 (75.0%)	80 (62.5%)	824 (63.0%)	
**Family History of Pancreatic Cancer**					<0.0001
Positive	28 (7.0%)	31 (4.9%)	13 (10.2%)	249 (19.0%)	
Negative	74 (18.5%)	125 (19.8%)	37 (28.9%)	235 (18.0%)	
Not provided	299 (74.6%)	475 (75.3%)	78 (60.9%)	824 (63.0%)	
**Pancreatitis panel results**					0.0002
Positive	113 (28.2%)	176 (27.9%)	18 (14.1%)	283 (21.6%)	
Negative	288 (71.8%)	455 (72.1%)	110 (85.9%)	1025 (78.4%)	

**Fig 1:**
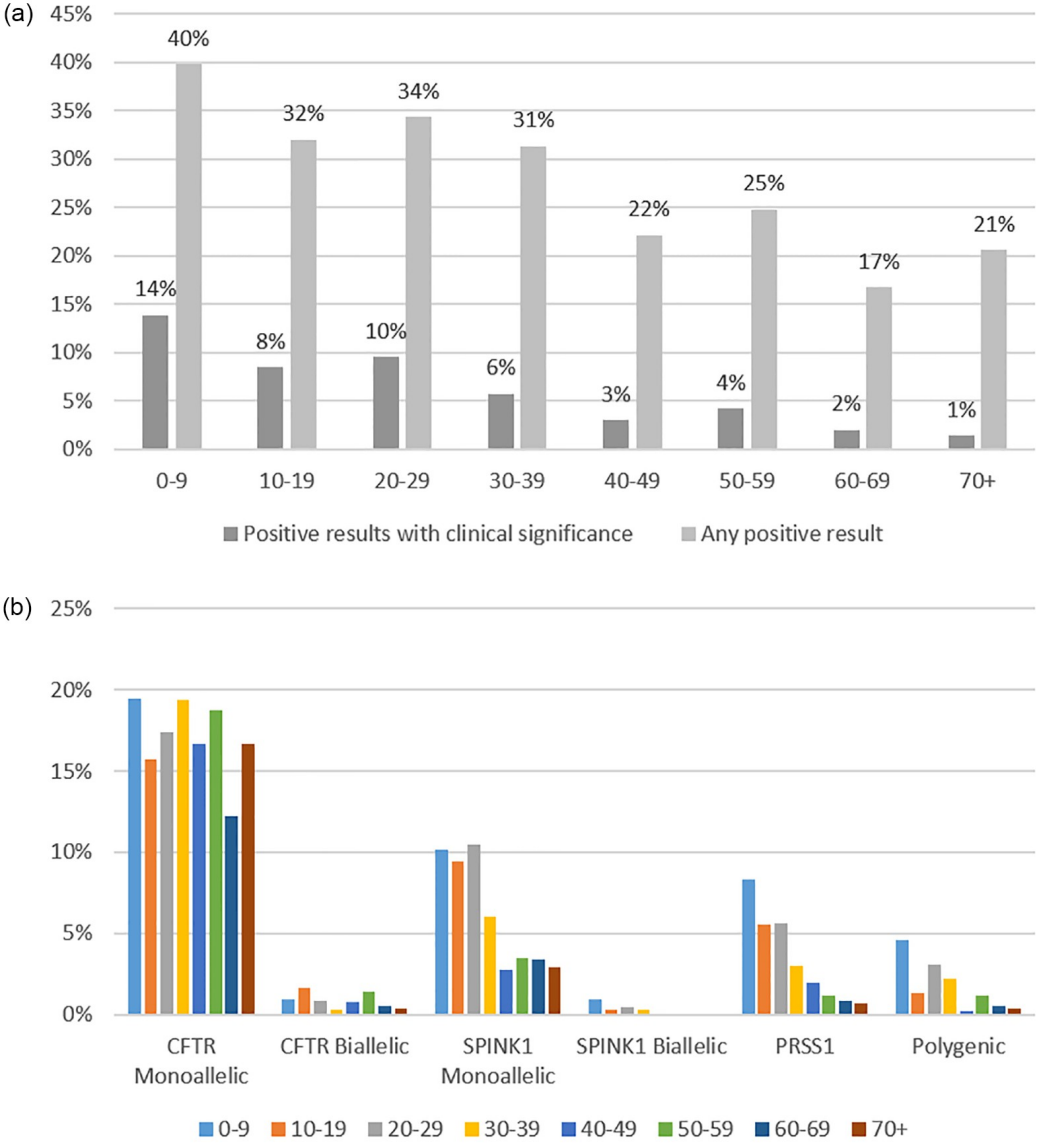
a: Prevalence of positive pancreatitis panel defined by any positive or positive with clinical implications according to age at the time of germline testing among subjects who completed the multigene pancreatitis panel from 2017 to 2022 (n = 2,468).b: Prevalence of PVs in pancreatitis-associated genes* according to age at the time of germline testing among subjects who completed the multigene pancreatitis panel from 2017 to 2022 (n = 2,468). *CASR, *CPA1*, and *CTRC* are not shown.

Overall, the most prevalent PVs were found in *CFTR* (16.9%), followed by *SPINK1* (5.4%) and *PRSS1* (2.8%) (Table 2). The prevalence of any positive result declined with age, but remained >15% at all age groups (Fig 1a). Similarly, the prevalence of a clinically significant result declined with age, but meaningful results were identified in all age groups. The prevalence of PVs in each gene varied with age, but monoallelic PVs in *CFTR* remained the most prevalent in all age categories (Fig 1b).

**Table 2:** Prevalence of pathogenic variants (PVs) in each pancreatitis-related gene according to the primary indication for testing.

	Acute Pancreatitis (n = 401)	Chronic Pancreatitis (n = 631)	Pancreatic Cancer (n = 128)	Other (n = 1308)	Overall (n = 2468)
**Any Clinically Significant Result** ^a^	32 (8.0%)	54 (8.6%)	0 (0%)	43 (3.4%)	129 (5.2%)
*PRSS1*	14 (3.5%)	32 (5.1%)	0 (0%)	22 (1.7%)	68 (2.8%)
* CFTR* biallelic	6 (1.5%)	7 (1.1%)	0 (0%)	8 (0.6%)	21 (0.9%)
* SPINK1* biallelic	1 (0.3%)	3 (0.5%)	0 (0%)	0 (0%)	4 (0.2%)
Polygenic^b^	10 (2.5%)	11 (1.7%)	0 (0%)	12 (0.9%)	33 (1.3%)
**Any Positive Result** ^a^	113 (28.2%)	176 (27.9%)	18 (14.1%)	283 (21.6%)	490 (19.9%)
* CASR*	0 (0%)	1 (0.2%)	0 (0%)	2 (0.2%)	3 (0.1%)
* CPA1*	0 (0%)	0 (0%)	0 (0%)	0 (0%)	0 (0%)
* CTRC*	6 (1.5%)	10 (1.6%)	2 (1.6%)	9 (0.7%)	27 (1.1%)
* CFTR* monoallelic	80 (20.0%)	114 (18.1%)	12 (10.2%)	209 (16.0%)	416 (16.9%)
* SPINK1* monoallelic	23 (5.7%)	48 (7.6%)	3 (2.3%)	58 (4.4%)	132 (5.4%)

^a^These rows represent total number of subjects with at least one positive result in the category. Some subjects may have multiple variants, so the sum of variants and subjects are not equal (i.e., a subject with both monoallelic *CFTR* and monoallelic *SPINK1* would be counted as *CFTR* monoallelic, *SPINK1* monoallelic, and polygenic but would only be counted once for prevalence calculations).

^b^Polygenic includes a combination of pathogenic variants in the following pancreatitis-related genes: *CTRC+SPINK1*, *CASR+SPINK1*, *CFTR+CTRC*, and *CFTR+SPINK1*.

### Acute pancreatitis

Among patients who underwent germline testing for a primary indication of AP, 28.2% (113/401) were found to have a PV in a pancreatitis-related gene and 8.0% (32/401) were found to have a clinically significant panel result (Table 2). Those with a PV were younger (30.1 vs 34.2 years, p = 0.04), but otherwise had similar sex, self-reported race/ethnicity, and family histories (S1 Table in S1 File).

### Chronic pancreatitis

Among patients who underwent germline testing for a primary indication of CP, 27.9% (176/631) were found to have a PV in a pancreatitis-related gene and 8.6% (54/631) were found to have a clinically significant panel result (Table 2). Those with a PV were more likely to have a positive family history of pancreatitis (20.5% vs 9.7%, p = 0.002), but otherwise had similar demographics (S2 Table in S1 File).

### Pancreatic cancer and other indications

Among patients who underwent germline testing related to pancreatic cancer or another indication, 14.1% (18/128) and 21.6% (283/1308) were found to have a PV in a pancreatitis-related gene. Clinically significant panel results were identified among 3.4% (43/1308) of subjects with another primary indication, but no clinically significant pancreatitis panel results were identified among subjects with pancreatic cancer (Table 2).

### Acute and chronic pancreatitis combined group

**Fig 2:**
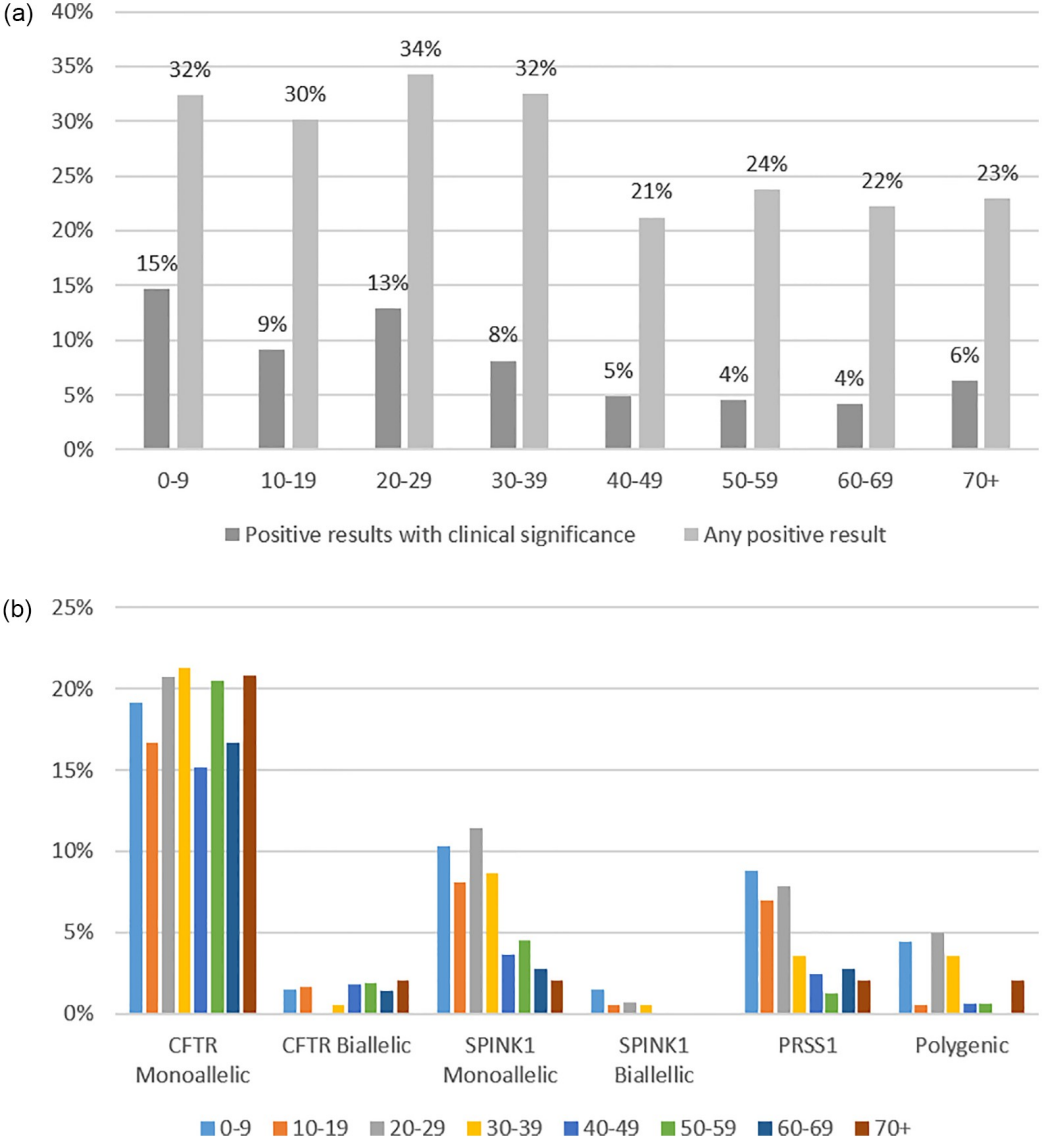
a: Prevalence of positive pancreatitis panel defined by any positive or positive with clinical implications according to age at the time of germline testing among subjects who completed the multigene pancreatitis panel for a primary indication of acute pancreatitis (AP) or chronic pancreatitis (CP) (n = 1,032). *CASR, *CPA1* and *CTRC* are not shown. b: Prevalence of PVs in pancreatitis-associated genes* according to age at the time of germline testing among subjects who completed the multigene pancreatitis panel for a primary indication of acute pancreatitis (AP) or chronic pancreatitis (CP) (n = 1,032). *CASR, *CPA1* and *CTRC* are not shown.

After excluding subjects with pancreatic cancer or other indication, individuals with AP and CP were considered together (n = 1,032) and the prevalence of any PV in pancreatitis-related genes was 28.0% (289/1,032) (Fig 2a and 2b). After adjusting for covariates, the multivariable model identified positive family history of pancreatitis as a significant predictor of a positive pancreatitis panel (Odds ratio (OR) 1.91, 95% confidence interval (CI): 1.10–3.32), and each 5-year increase in age as a significant negative predictor of a positive pancreatitis panel (OR 0.96, 95% CI: 0.92–1.00) (Table 3). When age was evaluated as a dichotomous covariate (≥35 vs <35 years), individuals who were ≥35 years old had 26% lower odds of having a positive pancreatitis panel than those younger than 35 years old (OR 0.74, 95% CI: 0.56–0.99, p = 0.043). Year of testing, sex, and family history of pancreatic cancer did not have significant associations with the outcome of positive pancreatitis panel result. An additional logistic regression was completed among 255 individuals with complete data, and the findings are similar (S3a, S3b Table in S1 File).

**Table 3:** Results of a multivariable model for the outcome of positive pancreatitis panel, including subjects with acute and chronic pancreatitis (n = 1,032) who underwent testing from 2017 to 2022.

Variable	OR	95% CI	p-value
**Age at Testing**			
5-year increase	0.959	0.922–0.997	0.0355
**Year of Testing**			
2017	Reference		
2018	0.61	0.35–1.08	0.0898
2019	0.71	0.42–1.20	0.2032
2020	0.64	0.38–1.07	0.0874
2021	0.75	0.45–1.26	0.2801
2022	0.53	0.24–1.19	0.1227
**Sex**			
Male	Reference		
Female	0.96	0.73–1.28	0.7912
**Primary Indication**			
AP	Reference		
CP	1.09	0.82–1.47	0.5489
**Self-Reported Race/Ethnicity**			
White	Reference		
Black/African American	0.98	0.53–1.82	0.9508
Asian	1.02	0.48–2.18	0.9662
More than one race	0.76	0.36–1.62	0.4808
Other	3.16	1.38–7.27	0.0067
Unknown/not provided	0.94	0.60–1.46	0.7758
**Family History of Pancreatitis**			
Negative	Reference		
Positive	1.91	1.10–3.32	0.0225
Not provided	1.07	0.30–3.78	0.9151
**Family History of Pancreatic Cancer**			
Negative	Reference		
Positive	0.71	0.35–1.44	0.3392
Not provided	0.85	0.24–3.06	0.8071

The prevalence of a clinically significant PV among the AP and CP combined group was 8.3% (86/1032). After adjusting for covariates, the multivariable model identified positive family history of pancreatitis as a significant predictor of a positive clinically significant pancreatitis panel (OR 8.59, 95% CI: 2.92–25.25), and each 5-year increase in age as a significant negative predictor of a positive pancreatitis panel (OR 0.89, 95% CI: 0.83–0.95) (Table 4). When age was evaluated as a dichotomous covariate (≥35 vs <35 years), individuals who were ≥35 years old had 56% lower odds of having a clinically significant panel result than those younger than 35 years old (OR 0.44, 95% CI: 0.27–0.71, p<0.001).

**Table 4:** Results of a multivariable model for the outcome of clinically meaningful positive pancreatitis panel, including subjects with acute and chronic pancreatitis who underwent testing from 2017 to 2022 (n = 1,032).

Variable	OR	95% CI	p-value
**Age**			0.0007
5-year increase	0.89	0.83–0.95	
**Sex**			
Male	Reference		
Female	0.92	0.58–1.45	0.7275
**Primary Indication**			
AP	Reference		
CP	1.23	0.76–2.00	0.4012
**Race Category**			
White	Reference		
Non-White	0.48	0.21–1.10	0.0817
Not provided	0.95	0.46–1.93	0.8752
**Family History of Pancreatitis**			
Negative	Reference		
Positive	8.59	2.92–25.25	<0.0001
Not provided	1.80	0.63–5.15	0.2722

## Discussion

In this report, we describe the results of germline testing with a multigene pancreatitis panel in the largest study population to date. Among patients with pancreatitis referred for genetic testing, monoallelic *CFTR* PVs were most frequently identified followed by monoallelic *SPINK1*. The most frequently identified clinically significant finding was a *PRSS1* PV. While individuals under 35 years old had a higher prevalence of overall PVs and clinically significant PVs, a meaningful number of subjects older than 35 years with pancreatitis had positive results. Based on these data, we suggest that genetic counseling and germline genetic testing should be recommended for subjects under age 35 with unexplained AP or CP and should be considered for subjects older than 35 years of age.

Prior to this report, the largest series of subjects with CP or recurrent AP undergoing germline genetic testing was reported by the North American Pancreatitis Study 2 (NAPS2) group. In their study, the prevalence of any PV in *CFTR*, *CTRC*, *PRSS1*, or *SPINK1* was 21% in CP and 26% in recurrent AP [14]. Our series reported the prevalence to be slightly higher (27.9% and 28.2%, respectively), which may be attributed to the use of full gene sequencing in our study compared to the multiplex single nucleotide polymorphism (SNP) array used in the NAPS2 study, or may be due to referral bias in our series [13]. Although the clinical features of AP and CP are not robustly described in our series, our multivariable models including age at the time of test completion provides new insight into the yield of germline testing for adults of varying ages with AP or CP.

We found a very low prevalence of PVs in *CASR* and *CPA1* among individuals in our series, which is similar to prior studies [29, 30]. Although PVs in *CASR* and *CPA1* have been identified as pancreatitis risk factors in some patients, the majority of described variants in these genes provide a minor contribution to the pathogenesis of CP [31, 32]. Variants in other possible pancreatitis-related genes, including *CEL*, *CLDN2*, *GGT1*, *MORC4*, *PRSS3*, *SBDS*, *SLC26A9*, *TRPV6*, and *UBR1*, may provide an explanation for otherwise unexplained AP or CP in some subjects but the significance of many variants have not been fully characterized to date [5]. Regarding specific variants in *CFTR*, the poly 5T allele was considered to be pathogenic in our study. This is consistent with a recent study suggesting a higher prevalence of idiopathic CP among individuals harboring a *CFTR* poly 5T allele [33]. Additionally, although the poly 5T allele is not considered to be causative of cystic fibrosis (CF), a number of non-CF causing PVs have been shown to be sufficient to contribute to the development of CP [34–36]. Finally, it is important to note that some PVs have a strong effect on pancreatitis risk and can be considered causative of pancreatitis, such as *PRSS1* R122H, while other PVs have lesser effects on pancreatitis risk and should be considered pancreatitis risk genes, such as heterozygous *CFTR* F508del or *SPINK1* N34S [37]. Thus, not all positive results should be considered equivalent and the genetic results should be interpreted in light of other additive risk factors.

Presently, society guidelines regarding germline genetic testing for patients with pancreatitis are based on expert opinion [16]. Thus, it is important to consider the benefits of genetic testing relative to other investigations in the evaluation of idiopathic pancreatitis. For comparison, a recent systematic review found the diagnostic yield of endoscopic ultrasound (EUS) in idiopathic AP to be 59%, with biliary etiologies found in 30%, CP identified in 12%, and neoplasms found in 2% [38]. Thus, an explanation for AP was provided in 12% (CP), and a direction for further management was provided in 32% (biliary and neoplasm). We show that germline testing detects a PV in 23.9% of subjects with AP or CP, including *PRSS1* (4.5%) and biallelic *CFTR* (1.3%) which have direct implications for management (pancreatic cancer screening and cascade testing for *PRSS1*, testing for cystic fibrosis for biallelic *CFTR*). An additional 2.4% with either biallelic *SPINK1* or multiple PVs might be considered for total pancreatectomy with islet autotransplantation in certain clinical scenarios. Thus, at present, germline testing provides insight to the etiology of pancreatitis in 23.9% and provides a direction for further management in 8.0%. When a direction for management is not identified, other benefits still include providing information and potentially reassurance for some patients, preventing more invasive diagnostic testing, providing opportunity for pre-conception counseling for recessive conditions (*CFTR*), and reinforcing lifestyle changes (e.g., smoking cessation) [16].

At the present time, additional precision medicine treatments are limited to clinical studies, but we speculate that some novel therapies will be available for clinical practice in the coming years. These developments will expand the value of germline genetic testing by recharacterizing some PVs as targetable by precision therapies. For example, among individuals with hereditary pancreatitis associated with *PRSS1*, a pilot study suggested clinical benefit from treatment with amlodipine [39]. More recently, CFTR modulating agents have been shown to reduce AP episode frequency and improve pancreatic exocrine function among individuals with cystic fibrosis [40, 41]. Case reports suggest that CFTR modulating agents may improve pancreatic manifestations among individuals with CFTR-related disorders, but larger studies are necessary [42, 43]. As precision medicine approaches are developed, agents correcting the detrimental effects of PVs in the most prevalent pancreatitis-associated genes should be prioritized in order to provide the greatest benefits for subjects with pancreatitis.

There are several limitations to consider when interpreting our findings. First, the study population has an unmeasured degree of selection bias due to the inclusion of patients referred for germline genetic testing. This bias could overstate the prevalence of PVs in the broader patient population. However, the large patient pool in this study and the wide range of referring practices should reduce the risk of this bias. Common to all secondary analyses of genetic testing datasets, the clinical annotation for each case is limited to a simplified test requisition form. This does not include specific and relevant details related to age at onset of pancreatitis and presence of other risk factors for pancreatitis. Furthermore, certain data was not provided on many requisition forms which limited the sample size for several analyses. In particular, indication for testing was not clearly AP, CP, or pancreatic cancer for 1,308 subjects, which limits the conclusions which can be drawn from this group. Because of this, individuals with an unclear indication for testing were excluded from the multivariable models. In addition, family history of pancreatitis or pancreatic cancer was not provided for many individuals. Accordingly, the data for these individuals was handled as “unknown/not provided” in the primary analysis (Table 3) and individuals were excluded in a sensitivity analysis (S3a, 3b Table in S1 File) and the findings were unchanged. Although the frequency of incomplete data limits the ability to apply our findings to individual scenarios, our data represents the real-world use of genetic testing and our results are useful for informing risk assessment during genetic counseling. Additional cross-sectional studies among well-phenotyped cohorts without selection bias will be necessary to confirm our findings.

Strengths of our study include the large sample size, which includes a wide variety of geographic regions, ages, and race/ethnicities which improve the generalizability of our findings. This large sample size also permits multivariable modeling, which we used to assess the independent contribution of age on the likelihood of identifying a PV. Additionally, the next-generation sequencing methodology included full gene sequencing and deletion/duplication analysis, which permits the identification of rare variants, single nucleotide substitutions, and copy-number variants, which would be missed with single nucleotide polymorphism analysis of only previously described variants. Finally, this sample included subjects referred for multiple indications, which provides insight into the genetic risk factors across the spectrum of pancreatic disease.

## Conclusion

In this study population, we demonstrate a high prevalence of PVs among patients undergoing multigene pancreatitis genetic testing. The yield is highest in younger individuals with a positive family history of pancreatitis and supports current recommendations for testing in patients with pancreatitis under age 35. In addition, we demonstrate clinically meaningful results in 4.7% of adults aged 50 years and older. This suggests that it may be reasonable to offer genetic counseling and germline testing to patients of all ages with idiopathic pancreatitis. Finally, we describe the relative prevalence of PVs in each pancreatitis-associated gene which may help prioritize the development of precision medicine therapeutic approaches.

## Supporting information

S1 File Pancreatitis multigene panel testing supplemental content. (DOCX)
