# Supplementary material for: Germline multigene panel testing in acute and chronic pancreatitis
Source: PLoS One. 2024 Aug 22;19(8):e0307076. doi: 10.1371/journal.pone.0307076 (PMC11341018; doi:10.1371/journal.pone.0307076)
Supplement: S1 File — (DOCX) [file pone.0307076.s001.docx]

**Title:** Germline Multigene Panel Testing in Acute and Chronic Pancreatitis

**Supporting Information**

**Supplementary Table 1:** Demographics and characteristics of subjects with acute pancreatitis who completed germline testing with a multigene pancreatitis panel from 2017 to 2022.

| **Variable** | **Positive Pancreatitis Panel**  **(n=113)** | **Negative Pancreatitis Panel**  **(n=288)** | **P-value** |
| --- | --- | --- | --- |
| **Sex** |  |  | 0.1926 |
| Female | 57 (50.44%) | 166 (57.64%) |  |
| Male | 56 (49.56%) | 122 (42.36%) |  |
| **Year of Testing** |  |  | 0.4804 |
| 2017 | 14 (12.39%) | 25 (8.68%) |  |
| 2018 | 13 (11.50%) | 37 (12.85%) |  |
| 2019 | 23 (20.35%) | 50 (17.36%) |  |
| 2020 | 23 (20.35%) | 76 (26.39%) |  |
| 2021 | 36 (31.86%) | 81 (28.13%) |  |
| 2022 | 4 (3.54%) | 19 (6.60%) |  |
| **Age at Testing** |  |  | 0.0410 |
| Mean (SD) | 30.14 (16.30) | 34.17 (18.12) |  |
| Median (IQR) | 30 (17-39) | 34.5 (18-48) |  |
| (min, max) | 3-72 | 1-77 |  |
| **Age (binary)** |  |  | 0.0466 |
| <35 years | 69 (61.06%) | 144 (50.00%) |  |
| ≥35 years | 44 (38.94%) | 144 (50.00%) |  |
| **Race Category** |  |  | 0.0547 |
| White | 81 (71.68%) | 221 (76.74%) |  |
| Black/AA | 8 (7.08%) | 14 (4.86%) |  |
| Asian | 6 (5.31%) | 11 (3.82%) |  |
| More than one race | 3 (2.65%) | 11 (3.82%) |  |
| Other | 10 (8.85%) | 7 (2.43%) |  |
| Unknown/Not provided | 5 (4.42%) | 24 (8.33%) |  |
| **FHX Pancreatitis** |  |  | 0.4268 |
| Positive | 19 (16.81%) | 35 (12.15%) |  |
| Negative | 13 (11.50%) | 40 (13.89%) |  |
| Not provided | 81 (71.68%) | 213 (73.96%) |  |
| **FHX Pancreatic Cancer** |  |  | 0.1122 |
| Positive | 4 (3.54%) | 24 (8.33%) |  |
| Negative | 26 (23.01%) | 48 (16.67%) |  |
| Not provided | 83 (73.45%) | 216 (75.00%) |  |
| **Other panel results** |  |  | 1.0000 |
| Positive | 3 (2.65%) | 7 (2.43%) |  |
| Negative | 110 (97.35%) | 281 (97.57%) |  |

**Supplementary Table 2:** Demographics and characteristics of subjects with chronic pancreatitis who completed germline testing with a multigene pancreatitis panel from 2017 to 2022.

| **Variable** | **Positive Pancreatitis Panel (n=176)** | **Negative Pancreatitis Panel**  **(n=455)** | **P-value** |
| --- | --- | --- | --- |
| **Sex** |  |  | 0.6279 |
| Female | 107 (60.80%) | 267 (58.68%) |  |
| Male | 69 (39.20%) | 188 (41.32%) |  |
| **Year of Testing** |  |  | 0.9035 |
| 2017 | 19 (10.80%) | 36 (7.91%) |  |
| 2018 | 27 (15.34%) | 78 (17.14%) |  |
| 2019 | 40 (22.73%) | 108 (23.74%) |  |
| 2020 | 44 (25.00%) | 115 (25.27%) |  |
| 2021 | 39 (22.16%) | 98 (21.54%) |  |
| 2022 | 7 (3.98%) | 20 (4.40%) |  |
| **Age at Testing** |  |  | 0.0626 |
| Mean (SD) | 35.97 (18.91) | 39.14 (19.23) |  |
| Median (IQR) | 35.5 (20-50.5) | 40 (23-53) |  |
| (min, max) | 0-87 | 2-84 |  |
| **Age (binary)** |  |  | 0.0590 |
| <35 years | 85 (48.30%) | 182 (40.00%) |  |
| ≥35 years | 91 (51.70%) | 273 (60.00%) |  |
| **Race Category** |  |  | 0.6430 |
| White | 124 (70.45%) | 312 (68.57%) |  |
| Black/AA | 7 (3.98%) | 28 (6.15%) |  |
| Asian | 4 (2.27%) | 15 (3.30%) |  |
| More than one race | 7 (3.98%) | 19 (4.18%) |  |
| Other | 4 (2.27%) | 4 (0.88%) |  |
| Unknown/Not provided | 30 (17.05%) | 77 (16.92%) |  |
| **FHX Pancreatitis** |  |  | 0.0016 |
| Positive | 36 (20.45%) | 44 (9.67%) |  |
| Negative | 19 (10.80%) | 59 (12.97%) |  |
| Not provided | 121 (68.75%) | 352 (77.36%) |  |
| **FHX Pancreatic Cancer** |  |  | 0.0577 |
| Positive | 10 (5.68%) | 21 (4.62%) |  |
| Negative | 45 (25.57%) | 80 (17.58%) |  |
| Not provided | 121 (68.75%) | 354 (77.80%) |  |
| **Other panel results** |  |  | 0.0694 |
| Positive | 0 (0.00%) | 10 (2.20%) |  |
| Negative | 176 (100.00%) | 445 (97.80%) |  |

**Supplemental Table 3a:** Results of a multivariable logistic regression model for the outcome of positive pancreatitis panel, including individuals with acute and chronic pancreatitis who underwent testing from 2017 to 2022 and had complete requisition forms (n=255)

| **Variable** | **OR** | **95% CI** | **p-value** |
| --- | --- | --- | --- |
| **Age**  1-year increase  5-year increase | 0.978  0.89 | 0.961-0.995  0.82-0.97 | 0.0105 |
| **Sex**  Male  Female | Reference  1.22 | 0.69-2.16 | 0.4908 |
| **Diagnosis Cohort**  AP  CP | Reference  1.52 | 0.85-2.71 | 0.1573 |
| **Race Category**  White  Black/African American  Other | Reference  1.41  1.17 | 0.39-5.11  0.51-2.69 | 0.5996  0.7092 |
| **Family history of pancreatitis**  Negative  Positive | Reference  1.79 | 1.00-3.21 | 0.0502 |
| **Family history of pancreatic cancer**  Negative  Positive | Reference  0.73 | 0.35-1.53 | 0.4083 |
| **Year of Testing*** | 1.18 | 0.97-1.44 | 0.0940 |

**Year of testing was considered as a continuous variable.*

**Supplemental Table 3b:** Results of a multivariable logistic regression model for the outcome of positive pancreatitis panel, including individuals with acute and chronic pancreatitis who underwent testing from 2017 to 2022 and had complete requisition forms (n=255)

| **Variable** | **OR** | **95% CI** | **p-value** |
| --- | --- | --- | --- |
| **Age ^**  < 35  35 or older | Ref  0.46 | 0.26-0.82 | 0.0078 |
| **Sex**  Male  Female | Reference  1.21 | 0.68-2.13 | 0.5218 |
| **Diagnosis Cohort**  AP  CP | Reference  1.46 | 0.82-2.60 | 0.1957 |
| **Race Category**  White  Black/African American  Other | Reference  1.54  1.20 | 0.42-5.56  0.52-2.76 | 0.5135  0.6713 |
| **Family history of pancreatitis**  Negative  Positive | Reference  1.79 | 1.00-3.21 | 0.0509 |
| **Family history of pancreatic cancer**  Negative  Positive | Reference  0.72 | 0.35-1.50 | 0.3853 |
| **Year of testing*** | 1.17 | 0.96-1.42 | 0.1161 |

*^ Age was considered as a binary variable.*

**Year of testing was considered as a continuous variable.*
